# Establishing Standards to Evaluate the Impact of Integrating Digital Health into Health Systems

**DOI:** 10.9745/GHSP-D-18-00230

**Published:** 2018-10-10

**Authors:** Alain Labrique, Lavanya Vasudevan, William Weiss, Kate Wilson

**Affiliations:** a Johns Hopkins Bloomberg School of Public Health, Baltimore, MD, USA.; b Department of Community and Family Medicine, Duke University School of Medicine, Durham, NC, USA.; c Center for Health Policy and Inequalities Research, Duke Global Health Institute, Durham, NC, USA.; d Digital Impact Alliance, Washington, DC, USA.

## Abstract

The key milestones in the rise of digital health illustrate efforts to bridge gaps in the evidence base, a shifting focus to scale-up and sustainability, growing attention to the precise costing of these strategies, and an emergent implementation science agenda that better characterizes the ecosystem—the social, political, economic, legal, and ethical context that supports digital health implementation—necessary to take digital health approaches to scale.

## INTRODUCTION

The rapid and global growth of mobile phone use in the last decade has enabled health system and development innovators to leverage digital health strategies in low-resource settings to alleviate persistent health system challenges. From supply chain management to frontline health-worker training, digital strategies have demonstrated varying degrees of promise. Despite the pervasiveness of these digital innovations, there has been rampant criticism of limited evidence to support their effectiveness.^1^ Numerous systematic reviews have been conducted with the same conclusion—the available evidence is of low-to-moderate quality and rigorous methodologies are needed to evaluate digital health strategies in low-resource settings.^2^ Despite this evidence deficit, global stakeholders' interest in implementing and scaling digital health strategies in these settings remains strong.^3^^,^^4^ In this commentary, we summarize the key milestones in the rise of digital health, illustrating efforts to bridge gaps in the evidence base, a shifting focus to scale-up and sustainability, growing attention to the precise costing of these strategies, and an emergent implementation science agenda to better characterize the necessary ecosystem of scale—the social, political, economic, legal, and ethical context that supports digital health implementation.^5^ We also identify key remaining gaps in the evaluation of digital health interventions to support their integration into health systems at scale.

## DISCORDANT PROLIFERATION: “PILOTITIS” AND FRUSTRATION

**FIGURE 1:**
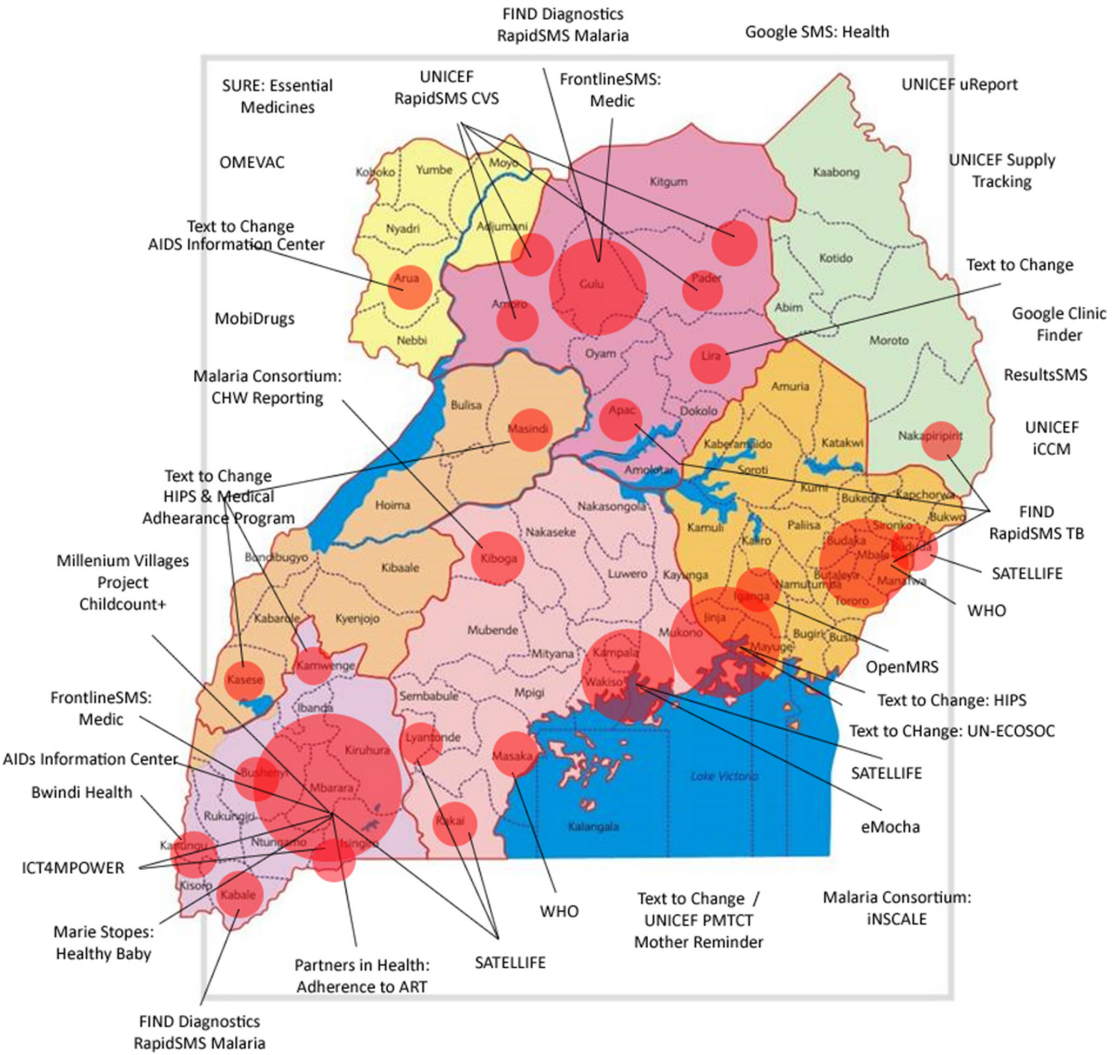
Map of Digital Health Pilot Projects in Uganda in 2010 Source: Sean Blaschke, UNICEF, written communication, May 2016.

In the early years of the mobile phone revolution, between about 2005 and 2010, the digital-health landscape was populated by numerous small-scale demonstration and pilot projects across low-, middle-, and high-income countries.^6^ The focus of these limited-scale ‘proof-of-concept’ initiatives was often simply to demonstrate concept feasibility, with little consideration of what might be required to scale-up the intervention. Moving from hundreds of users or data points to millions requires technical capacity that is large enough to withstand the load of national-scale use, attain and maintain economic sustainability, and achieve interoperability with other systems. While the pilot approach successfully accelerated the introduction of technology and pace of innovation, and resulted in unprecedented global awareness and interest in the implementation of digital health strategies, it also led to the development of predominantly stand-alone systems that provided limited evidence on their impact on health systems. The now infamous diagram (Figure 1) of mobile health (mHealth) pilot projects in 2010 in Uganda shows the number and spread of potentially redundant digital health investments. At the time, there was little coordination or planning or means of sharing information between projects; the figure, thus, illustrates the state of disarray that was likely across most countries involved in digital health experimentation in the early 2010s. In Uganda, this situation led to a moratorium on digital health projects, as the Ministry of Health worked to strengthen the coordination of information and communications technology (ICT) investments being made by international nongovernmental organizations and to sharply reduce potentially duplicative efforts in this space. Other important factors that may have contributed to these failures include the lack of local technical resources and capacity within the government to absorb these programs. Around this same time, global efforts to align and standardize information systems were led by the now-closed Health Metrics Network—a global partnership focused on widening the traditional scope of disease-centric information systems to broader national health systems monitoring and building country capacity for data-driven decision making.^7^

## SCRUTINY AND RECOGNITION OF THE NEED FOR RIGOROUS EVIDENCE

Between 2008 and 2013, Free, Cole-Lewis, Tamrat, Whittaker, and several others performed pragmatic reviews of the scant literature in mHealth, which highlighted (1) substantial variability in the quality and completeness of published findings and (2) inadequate descriptions of interventions' technologies, modes of delivery, and doses.^8^^–^^14^ While these authors lamented the lack of robust research designs being used to measure impact, some questioned whether alternative evaluation strategies based in qualitative science might be more appropriate at earlier stages of mHealth development. Questions also arose as to whether the randomized controlled trial (RCT) itself is the appropriate gold standard to measure efficacy of rapidly evolving digital health technologies.^15^

Early review of mHealth literature revealed considerable differences in the quality and completeness of published studies and the technology described within them.

As the lack of evidence to support digital health strategies became evident through these reviews, United Nations organizations, international nongovernmental organizations, unilateral and multilateral donors, and research institutions began advocating for use of rigorous evaluation methodologies for this new field. One of the first major responses was the development of the Bellagio Statement on eHealth Evidence following a high-level meeting of experts in 2011.^16^ The statement cautioned that “to improve health and reduce health inequalities, rigorous evaluation of eHealth is necessary to generate evidence and promote the appropriate integration and use of technologies.”^16^ A similar caveat was noted by participants at a landmark workshop the same year on mHealth evidence hosted by the U.S. National Institutes of Health: “In a healthcare system already burdened with suboptimal outcomes and excessive costs, premature adoption of untested mHealth technologies may detract from, rather than contribute to, what is needed for true overall health improvement.”^15^

Among the earliest strategies to undergo stringent evaluation were RCTs of mobile phone short message service/text messages to improve adherence to antiretroviral drugs. These first rigorous studies by Lester et al. and Pop-Eleches et al. remain among the most cited in this field (>800 and >600 times, respectively), illustrating the value of methodologic rigor to influence policy.^17^^,^^18^ A 2013 systematic review recognized that RCTs in this complex, emergent space had to be augmented by mixed-methods research to adequately understand contextual factors that influence the digital strategy's implementation efficacy across populations.^19^ About 20% of the sources considered in the review were drawn from the non-peer-reviewed literature, highlighting the risk of publication bias that could limit the availability of research in a rapidly growing, novel field. Even in this early stage of understanding, the importance of context on the efficacy and impact of intervention was clear, highlighting themes that would re-emerge 5 years later to dominate the digital health conversation.

In 2013, Tomlinson et al. published a sharp critique of the field, noting the identification of hundreds of mHealth studies demonstrating little known efficacy or effectiveness.^1^ They underscored the generally poor quality of research and the lack of a unifying language or framework to guide this space. That same year, Johns Hopkins University researchers published a review of the state of evidence in this space, noting that numerous examples of high-quality research exploring the efficacy of digital interventions were being developed, including those using accepted, rigorous methods of evaluation, such as RCTs.^20^ Several systematic reviews of digital interventions have been published since, corroborating the increasing volume of high-quality evidence.^10^^,^^21^^,^^22^

## DEVELOPMENT OF COMMON FRAMEWORKS

As efforts to generate and synthesize evidence in digital health grew, a unifying language to classify digital health investments became necessary. In late 2010, WHO convened the mHealth and Technical Evidence Review Group (mTERG; 2011–2014), whose first task was to develop a detailed taxonomy for adoption by the digital health community.^23^ The absence of a standardized language with clear definitions of technologies, channels, services, and, most importantly, the combination of technologies to accomplish a health system process, or digital health strategy, made it very difficult to analyze and synthesize emergent literature. Further complicating matters, donors and governments could not differentiate projects using different terms to describe their work, which led to duplicative investments. Because innovators did not work together or share experiences or resources, projects often “reinvented the wheel.”

**FIGURE 2:**
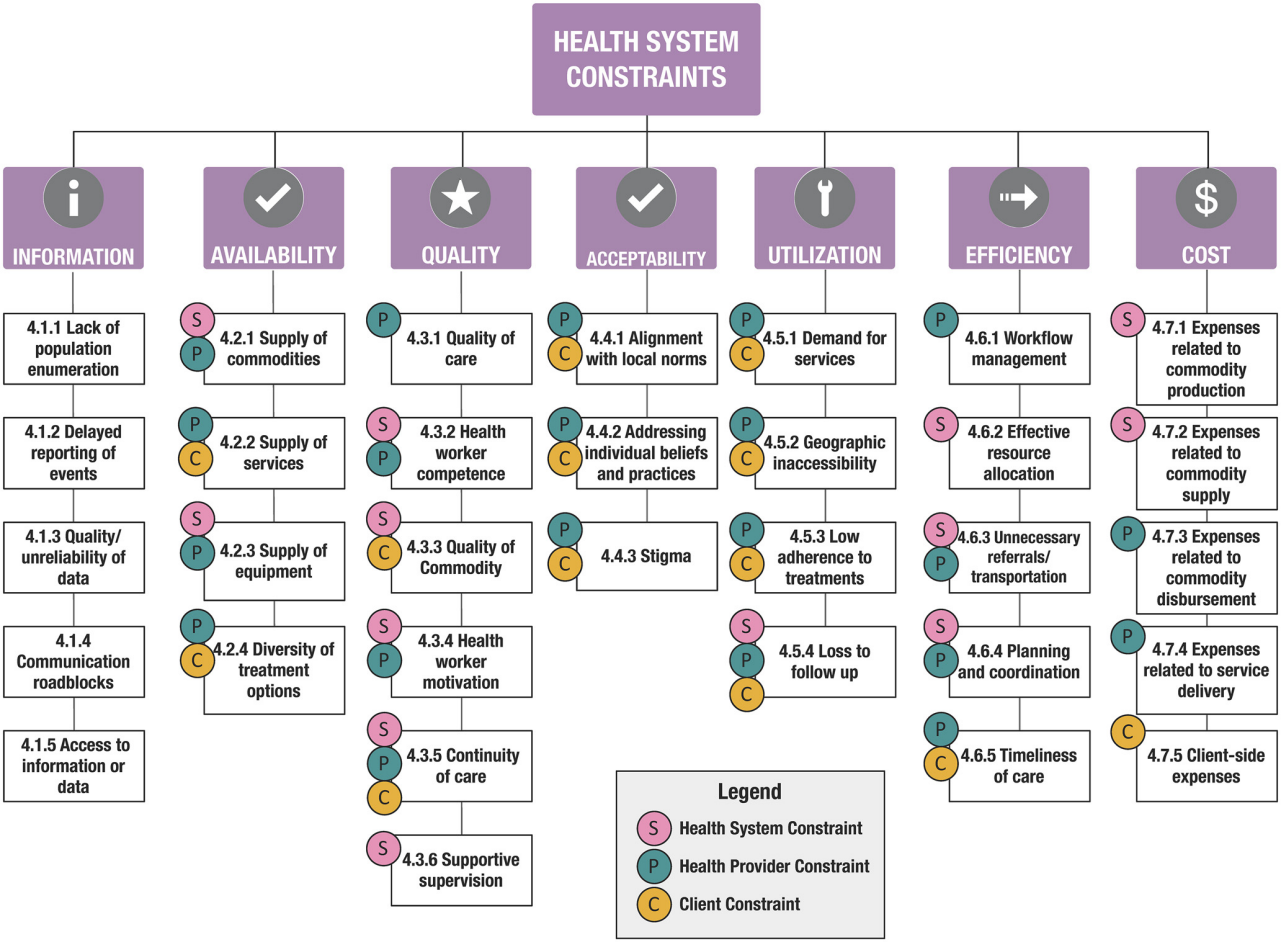
WHO Model Illustrating Health System Constraints Source: Mehl (2014).^26^

The “12 common applications” (or building blocks) framework,^24^ from WHO, the United Nations Children's Fund (UNICEF), the Johns Hopkins Bloomberg School of Public Health, and frog Design, is among the most widely used to describe projects in the emerging field of mHealth; it focuses on innovations that leverage mobile devices as a core component of its strategy. Since August 2013, the publication describing this visual framework has been downloaded over 56,000 times and cited 172 times. Building on a structure initially proposed by Mechael in 2010, this framework draws focus away from the technologies and toward the health system challenges they address.^25^ This framework was an effort to help digital health programs communicate the value of their innovations; to reduce duplicative efforts, as had happened in Uganda; and also to recognize that digital strategies should be considered health system process catalysts focused on overcoming constraints. This reframing, away from using technology for technology's sake, was useful for shifting attention to how digital tools could improve the quality or coverage of interventions of known efficacy. The goal of evaluations, therefore, the authors argued, should be less focused on health outcomes—such as vaccine-preventable morbidity or mortality—and more focused on the processes optimized by the digital catalyst, such as vaccine coverage or timeliness. As illustrated in Figure 2, WHO promoted a standard taxonomy of constraints to center discourse on the problems being solved by digital strategies—across layers of clients, providers, and the system—rather than the technologies themselves.^26^

The “12 common applications” framework focuses on the health system challenges digital technologies aim to address.

**FIGURE 3:**
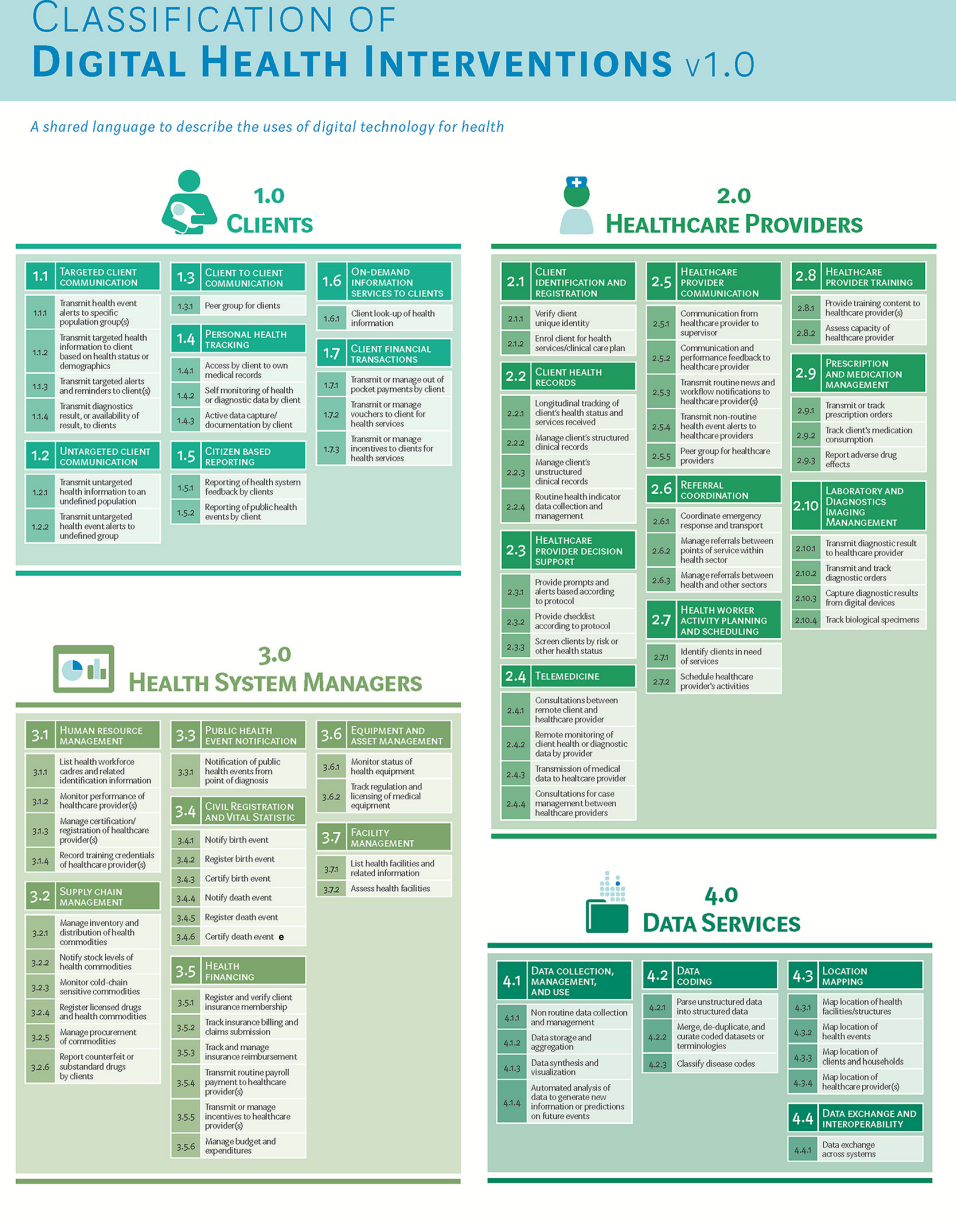
WHO Classification of Digital Health Interventions Released December 2017 Source: WHO (2018).^27^

In December 2017, after undertaking a 2-year process to update and standardize the taxonomy, WHO released a revised classification scheme for digital health interventions.^27^ Although more sophisticated technical frameworks have been adopted from architecture developed by Health Level Seven (HL7) or Control Objectives for Information and Related Technologies (COBIT),^28^^,^^29^ these frameworks are somewhat challenging for non-informaticians to access and integrate into public health discussions. Feedback from WHO mTERG, the Health Data Collaborative, and the wide community of practice led to the revised and extended standardized taxonomy to describe digital health interventions aligned to health systems challenges^27^ (Figure 3) in December 2017, which will be periodically updated by WHO to reflect the dynamic nature of the ecosystem.

In December 2017, WHO released a revised classification scheme for digital health interventions aligned to health systems challenges.

Among the frustrations also expressed by policy makers was the continued absence of demonstrated health impacts attributable to digital health investments. For most projects implemented in the early 2010s, digital health budgets were not commensurate with the effort required to set up efficacy and effectiveness studies or power them to detect health outcomes. Researchers demonstrated that through the intermediaries of coverage improvements modeling project outcomes could be used to prioritize digital health investments in cases where outcome measurement, such as infant or maternal mortality, might not be possible.^30^ To demonstrate this, they used the Lives Saved Tool (LiST),^30^ an evidence-based modeling software, to identify priority areas for maternal and neonatal health services in Bangladesh and Uganda. Their findings suggested that digital inputs targeting health system constraints—that reduced or limited skilled birth attendance and facility delivery—were able to increase coverage of both, potentially providing the highest impact to reducing mortality in the 2 countries. Together, the modeling approach and consequent digital health investment road map provided some guidance to seemingly uncoordinated investments in this space.

## IMPROVING THE QUALITY OF REPORTING DIGITAL HEALTH RESEARCH

As the use of shared language began to improve, several efforts to synthesize knowledge about archetypal digital strategies were undertaken. These efforts were soon frustrated by the wide variability in reporting quality across the work that had been published. In response, mTERG commissioned the development of mHealth evidence reporting and assessment (mERA) reporting guidelines in an effort to improve the completeness and comparability of mHealth reporting in peer-reviewed literature.^31^ The aim of these guidelines, now integrated within the EQUATOR (Enhancing the Quality and Transparency of Health Research) network^32^ of methodology-specific guidance, which includes PRISMA (Preferred Reporting Items for Systematic Reviews and Meta-Analyses) and CONSORT (Consolidated Standards of Reporting Trials), was to encourage authors to better describe the technologies and digital strategies they use as well as the implementation context. The mERA guidelines are recommended by WHO as a strategy to improve the synthesis of digital health research findings and improve replicability of interventions.

The mTERG commissioned the development of mERA reporting guidelines to improve the completeness and comparability of mHealth reporting in peer-reviewed literature.

**FIGURE 4:**
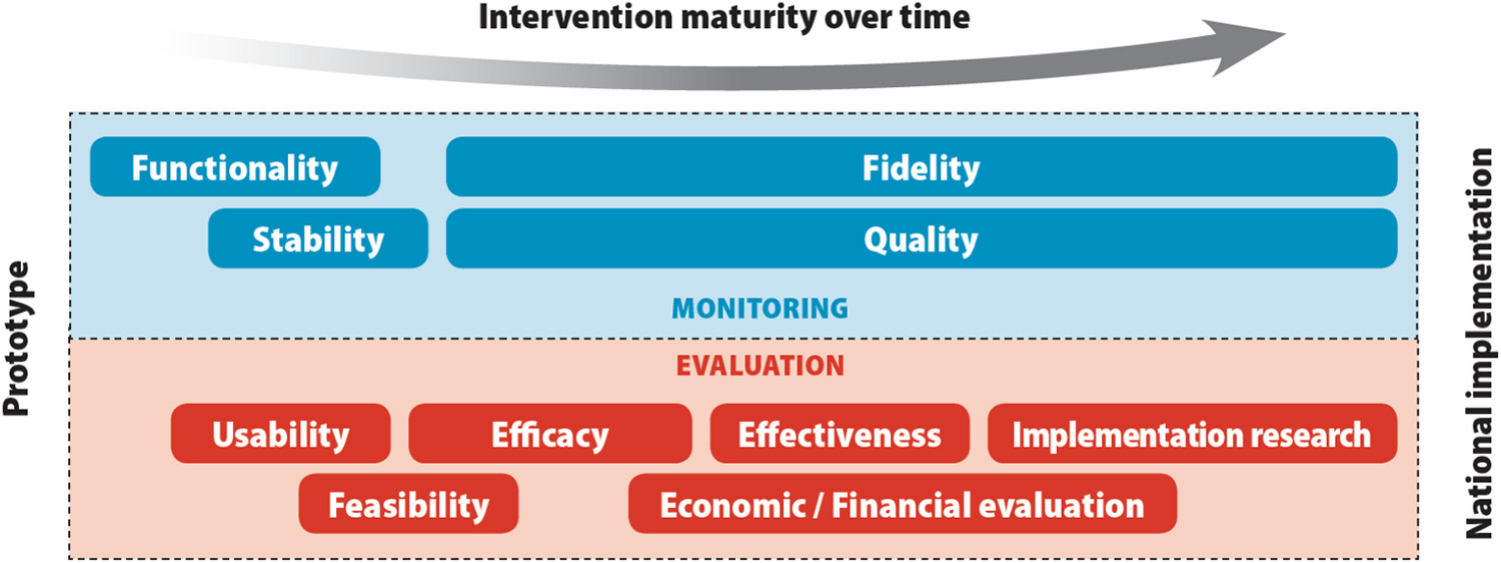
Methods and Objectives of Monitoring and Evaluation Activities Across the Lifespan of a Digital Health Program Source: World Health Organization (2016).^33^

In recent years, numerous parallel efforts toward strengthening the linkage between evidence generation and digital health scale-up have been implemented. In 2016, WHO published a practical guide to the monitoring and evaluation of digital health interventions.^33^ This guide, targeting implementers and researchers of digital health, prescribes a stage-based approach for testing digital health interventions—from feasibility and fidelity to impact evaluations (Figure 3). Several chapters of the guide are devoted to helping implementers and researchers tailor their evaluation programs with the objective of scale-up: from understanding stakeholder evidence needs for scale-up, formulating relevant objectives of the monitoring and evaluation plan, selecting thoughtful indicators that provide evidence to support program expansion, and ensuring the availability of reliable data sources for measurement of those indicators. The guide provides a diagram showing the methods and objectives of monitoring and evaluation activities used across the lifespan of a digital health program as it matures from prototype to national implementation (Figure 4).

Another important tool, *The MAPS* (mHealth Assessment and Planning for Scale) *Toolkit*^34^ was released in 2015 by WHO and partners to help strengthen the discourse about scaling-up digital health innovation. The toolkit integrates lessons from both failures and emerging successes from the digital health ecosystem, providing a semiquantitative approach to assessing program maturity and readiness for scale.^34^ The goal of this toolkit is to help project managers and other stakeholders periodically assess the maturity of their mHealth program and to provide stage-based strategies to bolster the potential for scale-up. This guide adapted and updated practical guidance from several field-tested WHO resources, including ExpandNet,^35^ thereby ensuring knowledge transfer of lessons and successful practices from other global health domains to digital health.

The aim of *The MAPS Toolkit* is to help project managers and other stakeholders assess their mHealth programs and to provide stage-based strategies to increase potential for scale-up.

## THE IMPORTANCE OF THE ENABLING ECOSYSTEM

While most of the resources discussed have focused on project-level introspection, *The MAPS Toolkit* focused some attention on the importance of the local environment in which digital health innovations are being tested. Despite substantial financial investments, several large projects had not succeeded in reaching or maintaining national scale, which led to the recognition that extrinsic factors play a crucial role in a program's survival.^36^^,^^37^ In 2011, WHO and the International Telecommunication Union released the *National eHealth Strategy Toolkit* in which they stated that “harnessing ICT for health requires strategic and integrated action at the national level, to make the best use of existing capacity while providing a solid foundation for investment and innovation.”^38^ The strategy toolkit has 3 core components: (1) development of the national eHealth vision, (2) development of an implementation road map, and (3) development of a plan to monitor and evaluate the implementation. This document was one of the earliest to place emphasis on stakeholder involvement—including the government—from the early stages of the planning and implementation process for digital health while focusing heavily on preparing the landscape to allow digital innovations to flourish. As a result, investments in large national and global programs like BBC Media Action's work with the government of India, the multi-partner Mobile Alliance for Maternal Action initiative, and the Better Immunization Data (BID) initiative were launched.^39^^–^^42^ Several scale-related guides were also developed during this time; for example, GSMA's interactive Service Maturity Tool^43^ was designed to help define the innovations, services, or features that appeal to different stakeholders, notably from the perspective of telecommunications partners, upon whose infrastructure most of these digital health strategies depend. The Program for Appropriate Technologies for Health (now PATH) identified the conditions of success required to establish and support digital health solutions and provided a framework for areas of investment in digital health solutions that are required to reach that level.^44^^,^^45^ Working with the ministries of health of Bangladesh, Ghana, and Tanzania, these tools were applied with a particular focus on improving the collection and management of health systems data to inform program planning, policy development, and resource allocation. The Tanzania framework and road map^45^ now serves as a useful illustration of how a systematic needs assessment process can (and should) be used to drive strategic investments in digital health.

## MOVING TOWARD HEALTH SYSTEM INTEGRATION: GAPS AND RECOMMENDATIONS

Despite a substantial increase in the level of organization and high-quality research in digital health, several key areas require more research. The transition to large-scale implementation has proven frustrating for implementing agencies, donors, and governments. While initial efforts to study digital health scale-up have yielded road maps and toolkits, such as *The MAPS Toolkit* and the monitoring and evaluation guide described earlier, there is an unmet need for high-quality economic evaluation and implementation science studies to better understand the complexities in scaling up digital innovation.^5^ We identified 4 key gaps in our quest to achieve health system integration of digital health strategies.

### Gap 1: Economic Evaluation of Digital Health Strategies

Governments need economic data to inform decisions on the adoption and scaling of digital health strategies. In the absence of economic data, governments lack the information to choose between competing digital health strategies, recognize the full value of individual strategies, or effectively plan and budget for the implementation of these strategies within their countries, when budgeting against other competing investments. In a recent systematic review of the economic evaluations of digital health strategies, two-thirds of the 39 studies conducted in middle- and high-income countries showed cost savings and increased cost-effectiveness resulting from the implementation of digital strategies.^22^ The study identified gaps in the evidence base for economic evaluations in low- and middle-income countries, the use of established reporting guidelines to improve quality of publications, and the selection of appropriate methods and indicators for evaluation to allow for meta-analysis. In 2017, Lefevre et al. published a 6-stage process for selecting and integrating economic and financial evaluation methods into the monitoring and evaluation of digital health strategies, with the goal of helping implementers understand the value of economic evaluations as a means to promote future efforts in this space.^46^ Funders of digital health need to require the systematic capture of economic data and the assessment of cost and benefits as part of the business case for scale-up.

### Gap 2: Enabling Ecosystem for Digital Health Strategies and Interventions

**FIGURE 5:**
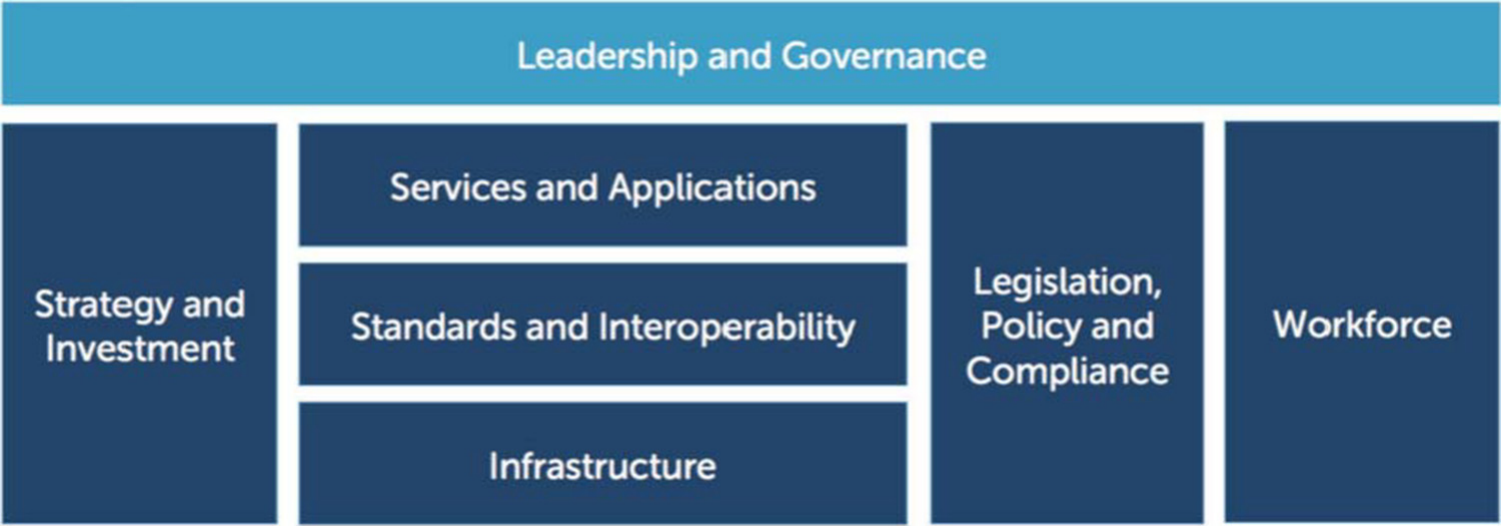
WHO-ITU National eHealth Strategy Toolkit eHealth Building Blocks Abbreviations: ITU, International Telecommunication Union; WHO, World Health Organization. Source: WHO and ITU (2012).^38^

Although many stakeholders have stressed the difficulty in expanding and sustaining digital programs at scale, we are only beginning to understand the necessary external factors required for success. In other words, the most effective ‘seed’ of innovation may not flourish if the ‘soil’ in which that seed is planted lacks the requisite nutrients to grow. This enabling ecosystem, as first described in the 2012 *National eHealth Strategy Toolkit*^38^ (Figure 5), includes the supporting technologies—such as electricity, network stability, and network capacity—necessary for systems to expand as well as the policies, governance structures, and human resources necessary to guide and manage the program as it grows. For example, the policy environment may need to include guidance for technical developers on data and interoperability standards, including what methods and practices should be used to store, transmit, and share data across platforms and maintain a high-level of security and confidentiality. A recent example of this type of standards is the South African *National Health Normative Standards Framework for Interoperability in eHealth*.^47^ Without such an enabling ecosystem, digital health solutions may not be sustainable and/or may continue to be siloed from other digital health investments. An implementation science approach is needed to better study and characterize these factors and learn from successful programs that currently exist at scale, such as MomConnect in South Africa^40^^,^^41^ or the Mwana Program in Zambia.^48^

Enabling ecosystems include the supporting technologies, policies, infrastructure, and human resources needed to support and expand key systems.

The 2017 Broadband Commission report on digital health^49^ focused on the importance of strengthening cooperation between ICT and health domains, largely through the actions of government leadership. Stressing the importance of a strong nation vision and strategy, the report provides multiple examples of road maps that have helped countries invest strategically, over time, in building health information system capacity and an enabling environment required for the success of these systems.^49^ Health information systems require careful, layered planning and implementation, including equal attention to and investment in the technical systems being put into place and the human and institutional change management required to adapt to the emerging status quo.

### Gap 3: Financial and Sustainability Evaluation

Inadequate focus on monitoring the quality of programs, once deployed, has led a number of large digital investments to have limited impact.^50^ Just as with non-digital projects, research and guidance on fidelity and quality must be part of implementation planning for long-term stability. Resources and improved tools must be developed to facilitate program monitoring—from system functionality to staff performance quality. Few projects, globally, have reached a level of scale or longevity needed to provide insight into the actual anticipated and unanticipated costs of large digital health operations. In contrast to many decades of well-documented operational costs for paper-based systems, program planners lack reliable information on the durability of digital assets, necessary overages, and contingency procurements to allow for digital device failures or losses. Models estimating the total cost of ownership or operational costs are often based on short-term programs or extrapolated from pilot and research environments, which may not accurately represent real-world data. Economies of scale and cost-savings possible through the use of shared digital assets remain underexplored. Finally, understanding the collateral gains to be made from digital investments can also strengthen the case for these investments; that is, the time and effort previously spent on manual data summarization or aggregation—often repeated at multiple health-system levels—can be liberated for repurposing to other primary-care tasks.

Research and guidance on fidelity and quality must be part of implementation planning in order to ensure long-term stability.

### Gap 4: Effective Pathways for Change Management and Data Use

Lastly, one of the more difficult challenges for digital health lies not in the development or distribution of technology, but in maximizing the use of data generated to improve system performance and, consequently, health outcomes. The data capture and population health tracking systems in use in many low- and middle-income countries have been entrenched for decades—they are deeply reliant on paper, with complex data aggregation and reporting systems in place. Understanding and addressing the threats to established processes, especially potential changes to transparency and accountability, are essential to future success. Strategies to develop cultures of data use are needed to shift the way systems are managed on quarterly or annual cycles to more frequent access of real-time data “on demand.” Recognizing and mitigating the perceived risks of better data—increased visibility of dysfunction, exposure of incompetence or graft, and poor program performance—is vital to the long-term success of these interventions. In several settings, the monitoring of real-time or near real-time program performance using national-level dashboards has become a crucial part of providing data for government operational planning and reporting. In Ghana, Kenya, Tanzania, Bangladesh, and at least 50 other countries, data entered into the District Health Information System 2 is being used to support data-driven decision making with active programs to verify and improve their quality of data reporting.^48^

**FIGURE 6:**
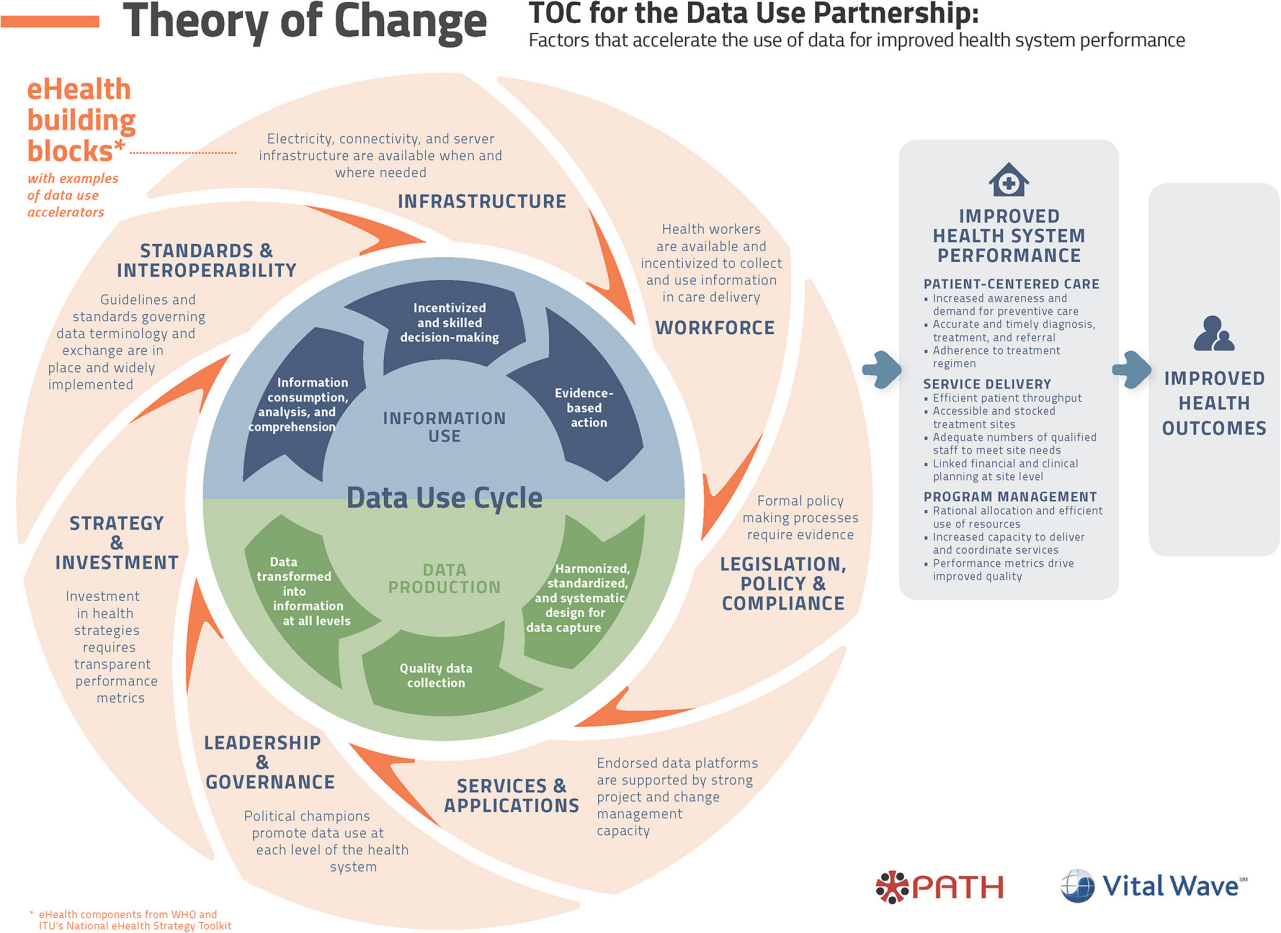
The PATH-Vital Wave Data Use Cycle Source: PATH (2016).^51^

The use of improved denominators, for example, rather than target population estimates may reveal a lower rate of coverage, which may mean perceived needs or gains are lower than expected. Supported by more accurate data, these issues can be overcome by strong leadership and development partners as they move toward results-based financing. In 2016, PATH and VitalWave released an elegant framework^51^ (Figure 6) illustrating how the digital health building blocks introduced in Figure 5 are interwoven into a data-use cycle where data production feeds into information use through a continuous feedback loop. Already overtaxed health systems may have difficulty finding resources, particularly skilled staff, to actively monitor data quality and take actions to improve it. Innovations that harness machine learning/artificial intelligence to identify errors or aberrations in data quality may help to alleviate this burden. More research is also needed to elucidate program components that promote data-driven decision making and evidence-based action.

## CONCLUSIONS

We suggest that intensification of efforts to bridge these gaps will likely alleviate some of the frustrations associated with scaling and sustaining digital health strategies. Throughout this paper, we have described how an increased push for evidence by donor agencies and global stakeholders has driven the growth of peer-reviewed literature describing the benefits of digital health for mitigating health system constraints. Looking ahead, a similar call to evidence for economic evaluation and adoption of an implementation science lens is crucial to driving health system integration. In 2016, WHO established a guidelines development group to assess current evidence and recommendations for digital strategies. The guidelines development process not only recommends appropriate strategies that are adequately supported by sufficient evidence but also highlights promising strategies that currently have a low threshold of evidence that require future research, with a particular eye toward health system integration of these strategies. For the evaluation of digital health strategies, the standards established during the guidelines development process should help countries facilitate streamlining the application of and investments in digital strategies, moving us closer to the vision of a health system of the future.

The process of systems change is difficult, especially when current practices are the result of decades of professional practices layered upon each other. Health systems are often massive bureaucracies with limited resources, struggling to provide essential population services to large numbers of clients. The introduction of digital health innovations is still seen by many as a wasteful distraction from core health system functions, potentially diverting resources from primary services. The failures of imperfect pilot systems and gaps between promised results and actual performance seem to vindicate these claims. However, the needs of a rapidly growing human population and the challenges of measuring progress toward and meeting global health goals requires taking important steps to improve health-system reporting. After an initial period of unchecked enthusiasm and technologic experimentation, the field of digital health is now structured and increasingly organized. The evidence base of digital health approaches that have been successfully scaled up is growing, and new technology and shared standards provide a framework that can decrease the risk and amplify the promises of digital health investments. Digital health innovations are increasing accessibility, promoting transparency, and have the capacity to increase accountability—all necessary facets of lasting health systems strengthening.

The growing evidence base of successful digital health approaches, new technologies, and shared standards provide a sound foundation for supporting digital health investments.

## Notes

**Peer Reviewed**

**Funding:** This work was made possible by a grant from the Aetna Foundation, with support from the World Health Organization and The Johns Hopkins University Global mHealth Initiative. In addition, Vasudevan's effort on this publication was supported in part by the National Center for Advancing Translational Sciences of the National Institutes of Health under Award Number 1KL2TR002554.
